# Rabies Virus Vector Transgene Expression Level and Cytotoxicity Improvement Induced by Deletion of Glycoprotein Gene

**DOI:** 10.1371/journal.pone.0080245

**Published:** 2013-11-07

**Authors:** Shinya Ohara, Sho Sato, Kei Oyama, Ken-Ichiro Tsutsui, Toshio Iijima

**Affiliations:** Division of Systems Neuroscience, Tohoku University Graduate School of Life Sciences, Sendai, Japan; Thomas Jefferson University, United States of America

## Abstract

The glycoprotein (G) of rabies virus (RV) is required for binding to neuronal receptors and for viral entry. G-deleted RV vector is a powerful tool for investigating the organization and function of the neural circuits. It gives the investigator the ability to genetically target initial infection to particular neurons and to control trans-synaptic propagation. In this study we have quantitatively evaluated the effect of G gene deletion on the cytotoxicity and transgene expression level of the RV vector. We compared the characteristics of the propagation-competent RV vector (rHEP5.0-CVSG-mRFP) and the G-deleted RV vector (rHEP5.0-ΔG-mRFP), both of which are based on the attenuated HEP-Flury strain and express monomeric red fluorescent protein (mRFP) as a transgene. rHEP5.0-ΔG-mRFP showed lower cytotoxicity than rHEP5.0-CVSG-mRFP, and within 16 days of infection we found no change in the basic electrophysiological properties of neurons infected with the rHEP5.0-ΔG-mRFP. The mRFP expression level of rHEP5.0-ΔG-mRFP was much higher than that of rHEP5.0-CVSG-mRFP, and 3 days after infection the retrogradely infected neurons were clearly visualized by the expressed fluorescent protein without any staining. This may be due to the low cytotoxicity and/or the presumed change in the polymerase gene (L) expression level of the G-deleted RV vector. Although the mechanisms remains to be clarified, the results of this study indicate that deletion of the G gene greatly improves the usability of the RV vector for studying the organization and function of the neural circuits by decreasing the cytotoxicity and increasing the transgene expression level.

## Introduction

Rabies virus (RV) selectively infects neurons and propagates between synaptically connected neurons in an exclusively retrograde direction [1-4]. We have previously developed a recombinant RV vector that was derived from an avirulent vector (rHEP3.0) based on the HEP-Flury strain [5,6]. This recombinant RV vector (rHEP5.0-CVSG) propagated trans-synaptically in a retrograde direction. Furthermore, the morphological features of the infected neurons were clearly visualized by using antibodies against the expressed marker protein, such as green fluorescent protein or β-galactosidase [7]. These features make this propagation-competent RV vector a useful trans-synaptic tracer. By using this viral vector, we have established a dual trans-synaptic tracing method that could be used to identify two different neuronal circuits in the same experiment and have revealed the organization of commissural connectivity in the hippocampus [7,8]. 

Wickersham et al. [9,10] have recently reported a G-deleted RV vector that is based on a SAD-B19 strain (SADΔG) and can be used to study the structure and function of neural circuits [11-14]. The envelope spike glycoprotein of RV is responsible for binding to receptors on the surface of a host cell and for viral entry. Since infectious virus particles that bear the glycoprotein cannot be produced by neurons infected with the G-deleted RV, this virus cannot propagate trans-synaptically from the initially infected neurons [15]. The advantage of deleting the G gene is that it gives the investigator the ability to genetically target infection to particular neurons and to their presynaptic neurons, which can be achieved by pseudotyping the virus and supplying the G gene within the initially infected neurons [13,16]. These infection features of the G-deleted virus enable a far more detailed understanding of the neural circuits.

**Figure 1:**
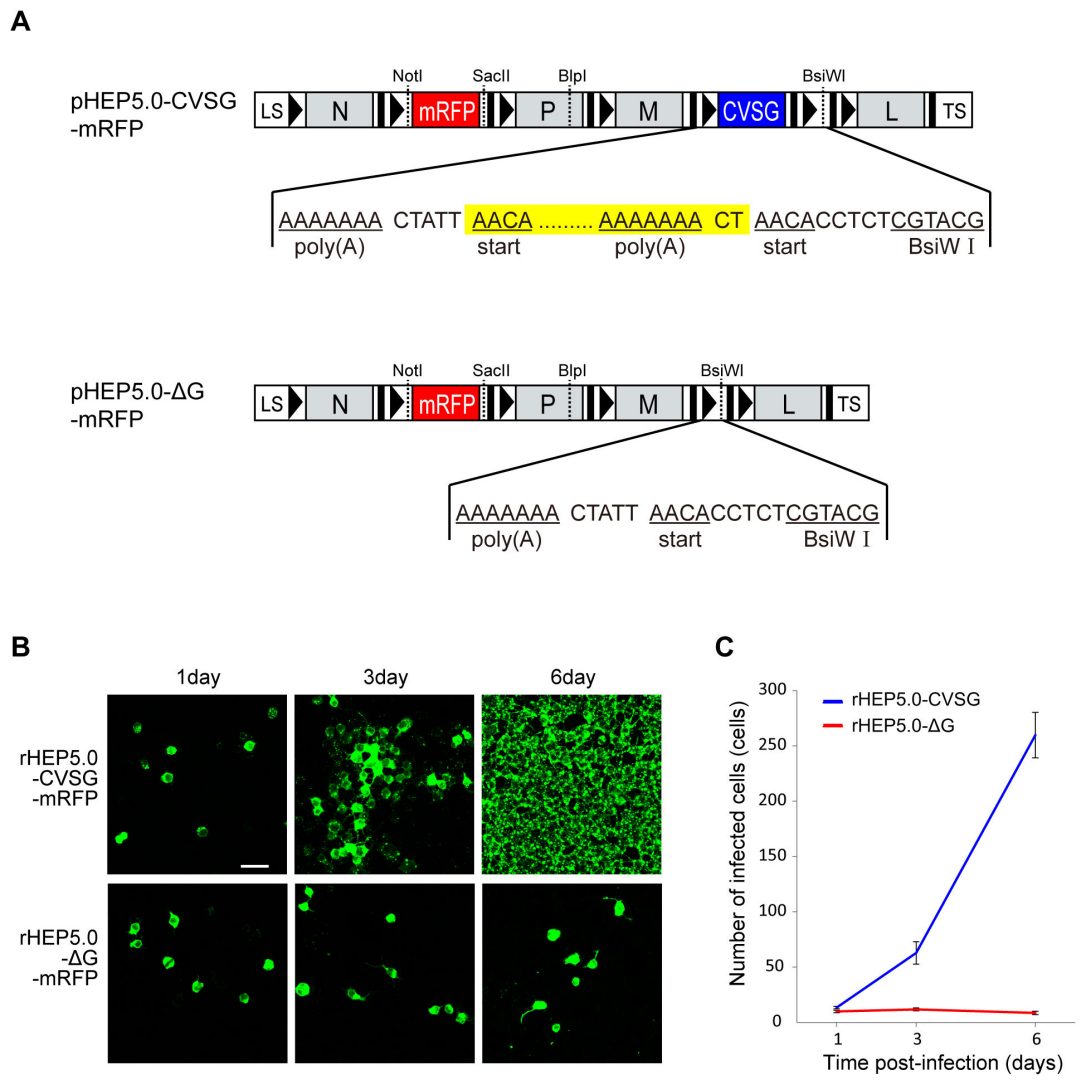
Genome structures and the spread of RV vectors in cultured cells. **A**: Genome organization of the recombinant RV vectors and the sequence from the polyadenylation signal of the M gene to the *Bsi* WI site in the additional transcription unit. The transcription start and stop/polyadenylation signals are respectively shown by black bars and black arrowheads in the schematic diagram and are underlined in the sequence. The propagation-competent RV vector (pHEP5.0-CVSG-mRFP) was based on the HEP-Flury strain except for the G gene, which was derived from the challenge virus standard strain (CVSG). This viral vector has two additional transcription units for foreign gene expression, one after the sequence coding the N gene and the other after the sequence coding the G gene. The gene for monomeric red fluorescent protein (mRFP) was inserted in the former additional transcription unit. In pHEP5.0-ΔG-mRFP, the whole transcription unit of the G gene and the 2-nucleotide intergenic region after the G gene (yellow shaded box) was deleted from pHEP5.0-CVSG-mRFP. LS, leader sequence; TS, trailer sequence. **B**: Fluorescence photomicrographs showing the spread of rHEP5.0-CVSG and rHEP5.0-ΔG infection at different days post-infection. Infected cells were visualized by the immunofluorescence of the N protein. Scale bar = 50 μm. **C**: Number of infected cells (mean ± SEM) for each strain at different post-infection times. Note that the number of rHEP5.0-CVSG-mRFP-infected cells increased with the post-infection time but the number of rHEP5.0-ΔG-mRFP-infected cells did not.

To improve the infection features of our RV vector, we have generated a G-deleted RV vector (rHEP5.0-ΔG) by deleting the entire G gene from the genome of rHEP5.0-CVSG. A monomeric red fluorescent protein (mRFP) was inserted into each vector (rHEP5.0-CVSG-mRFP and rHEP5.0-ΔG-mRFP, Figure 1A), and the characteristics of these two RV vectors were examined *in vitro* and *in vivo*. As we had expected, rHEP5.0-ΔG-mRFP did infect neurons retrogradely but did not propagate trans-synaptically. Furthermore, out of our expectation, the deletion of the G gene markedly affected aspects of the RV vector other than its infection properties such as the transgene expression level [17]. This may be due to two effects which could be induced by the deletion of the　G gene: (1) the reduction of cytotoxicity by the loss of G expression; (2) the presumed increase of the L gene expression. In this study we have quantitatively evaluated the enhancement of the transgene expression level and the reduction of the cytotoxicity that are due to the deletion of G gene. Although it remains unclear whether the loss of G expression itself causes the change in the transgene expression level, the results indicate that the deletion of G gene has large effects on the RV vector resulting in the alteration of its nature, and makes this vector much suitable for anatomical and physiological studies.

## Materials and Methods

### Plasmid construction and virus recovery

The G-deleted RV vector (rHEP5.0-ΔG) was constructed by deleting the entire G gene from pHEP5.0-CVSG (DDBJ/GenBank/EMBL accession number, AB839170). A PCR fragment containing the sequence from the *Blp* I site of the P gene to the 5-nucleotide intergenic region after the M gene was amplified using the following primers:

P-Blp-5: 5’- CAAGCTAAGCAAAATCATGCAAGATGA-3’ (the *Blp* I site is underlined)

BsiWI-M-3: 5’- AGTCGTACGAGAGGTGTTAATAGTTTTTTTCACATCCA -3’ (the *Bsi* WI site, transcription start signal, and polyadenylation signal are underlined in that order)

The sequence between the *Blp* I site and *Bsi* WI site was removed from pHEP5.0-CVSG and was replaced with the PCR fragment shown above. The resulting plasmid was designated pHEP5.0-ΔG. We have deposited the sequence of the full-length cDNA of pHEP5.0-ΔG in the DDBJ/GenBank/EMBL database (accession number, AB839169). In this study we used RV vectors that express mRFP as a transgene. mRFP cDNA containing the *Not* I site, open reading frame, and the *Sac* II site was amplified and inserted into the additional transcription insertion unit between the N and P genes of pHEP5.0-CVSG and the pHEP5.0-ΔG. The resulting plasmids were designated pHEP5.0-CVSG-mRFP and pHEP5.0-ΔG–mRFP (Figure 1A), and the complete sequences were deposited in the DDBJ/GenBank/EMBL database (accession numbers: pHEP5.0-CVSG-mRFP, AB855657; pHEP5.0-ΔG–mRFP, AB855650).

pHEP5.0-CVSG-mRFP and pHEP5.0-ΔG-mRFP were rescued by using mouse neuroblastoma cells of A/J mouse origin (NA) as described previously [6]. The rescued viruses generated from pHEP5.0-CVSG-mRFP and pHEP5.0-ΔG-mRFP were designated rHEP5.0-CVSG-mRFP and rHEP5.0-ΔG-mRFP, respectively. Since rHEP5.0-ΔG-mRFP does not express the glycoprotein, NA cell line expressing the RV glycoprotein was used for the inoculation of this virus. Both viral strains were concentrated using centrifugal filter devices (Amicon Ultra-15). A viral suspension was kept in small aliquots at −80°C. Each aliquot was thawed in a safety cabinet before each experiment. To determine the viral titer, we conducted a direct florescent test using NA cells as described elsewhere [18].

### Viral infection in cultured cells

NA cells were plated on glass coverslips in a 12-well plate and maintained at 37°C in minimum essential medium supplemented with 10% heat-inactivated fetal bovine serum (FBS). To evaluate the infection properties of rHEP5.0-CVSG-mRFP and rHEP5.0-ΔG-mRFP, each virus was applied to the dish at a multiplicity of infection (m.o.i) of 1. To evaluate the efficacy of mRFP expression of the two vectors, each of the two viruses was applied at a m.o.i of 15. One hour after infection the inoculum was replaced with fresh medium and cells were incubated at 34°C. The spread of the viruses and the fluorescence intensity of the mRFP were examined at different time points (1–9 days post-infection). Cells were fixed for 45 min at 4°C in phosphate buffered saline (PBS) containing 4% formaldehyde, washed with PBS three times, and then soaked for an hour at room temperature in PBS containing 5% goat serum and 0.1% Triton X-100. Cells were then incubated overnight at 4°C with monospecific rabbit anti-N antiserum [19] diluted in PBS containing 0.1% Triton X-100 and 0.025% NaN_3_. After the primary antibody was aspirated, cells were washed and permeabilized in PBS containing 0.1% Triton X-100 (PBT). Cells were then incubated for 2 hours at room temperature in Cy2-conjugated anti-rabbit goat IgG (1:300; Jackson ImmunoResearch, West Grove, PA) and Hoechst 33258 solution (1:1000; Dojindo, Japan) diluted in PBT. The cells were then washed 3 times with PBS, and coverslips were adhered to glass slides with mounting medium. Fluorescent labeling was assessed using the confocal laser-scanning microscope LSM 5 Exciter (Carl Zeiss). The fluorescence intensity of mRFP was quantified by measuring the average fluorescence intensity per pixel in the infected neurons with ImageJ software (http://rsb.info.nih.gov/ij). Since we measured the fluorescence intensity only from cells whose fluorescence was above the fixed threshold, apoptotic cells which hardly show fluorescence were not included in the analysis.

### Physiological recording *in vitro*

NA cells were plated on a poly-D-Lysine coated 35-mm dish with a coverslip bottom, and the cells were infected with each virus as described in the preceding paragraph. Two to 6 days later the medium was replaced with extracellular solution (150 mM NaCl, 5 mM KCl, 2.4 mM CaCl_2_, 1.3 mM MgCl_2_, 10 mM D-glucose, 10 mM HEPES, 0.1% BSA, adjusted to pH 7.4 with NaOH), and the dish was mounted on an inverted microscope (Axiovert 200M, Carl Zeiss). During recording, the bath solution was perfused with fresh extracellular solution at 34–37°C. Infected cells were detected by direct visual observation of mRFP fluorescence.

Patch-clamp recordings in the whole-cell mode were made using a patch-clamp amplifier with a capacitive headstage (Axoclamp 200B, Axon Instruments, USA) using glass recording electrodes (3–5 MΩ) filled with intracellular solution (140 mM potassium gluconate, 5 mM KCl, 1 mM MgCl_2_, 0.1 mM EGTA, 2 mM Mg-ATP, 5 mM HEPES, adjusted to pH 7.2 with KOH). Whole-cell recordings were low-pass-filtered at 1 kHz and digitized at 10 kHz. Data were digitized with a digitizer (Digidata 1342, Axon Instruments, USA) and fed into a computer for off-line analysis using AxoClamp 9.0 software (Axon Instruments, USA). 

### Viral infection *in vivo*

Young adult male Wistar rats weighing 200–230 g were used in this study. All experiments were approved by the Center for Laboratory Animal Research, Tohoku University, and were conducted according to the Guidelines of the National Institutes of Health and the Guidelines for Animal Care and Use published by our institute. We set clinical signs of rabies (slow and circular movements, paralysis, cachexia) as humane endpoints, but since no rats showed any clinical signs of rabies, all rats were sacrificed with an overdose of sodium pentobarbital after a certain survival time according to the experimental schedule. All rats infected with the propagation-competent RV vector (rHEP5.0-CVSG-mRFP) were sacrificed at relatively short survival times (within 7 days), whereas some of the rats infected with the G-deleted RV vector (rHEP5.0-ΔG-mRFP) were sacrificed after a longer survival time (21 days) because this viral vector is known to be non-pathogenic and does not cause any detectable symptoms [15]. All experiments with injections of recombinant RV vectors were carried out in a special laboratory (biosafety level 2) designated for *in vivo* infectious experiments.

Rats were deeply anaesthetized with ketamine (80.0 mg/kg, i.p.) and xylazine (0.8 mg/kg, i.p.), and were mounted in the stereotaxic frame. The skull was exposed, and a small burr hole was drilled above the injection site. The injection was made by means of a glass micropipette (tip diameter = 30 μm) connected to a 1-μl Hamilton microsyringe.

Either 800 nl of rHEP5.0-CVSG-mRFP (1.0×10^8^ FFU/ml, n = 5) or rHEP5.0-ΔG-mRFP (1.0×10^8^ FFU/ml, n = 8) was injected into the medial entorhinal cortex (MEC). Each virus was injected with 1% of pontamine sky blue so that the injection sites could be located. After the injection at 40 nl per minute, the pipette was left in place for an additional 15 minutes before it was withdrawn. The skin wound was sutured, and the animal was monitored for recovery from anesthesia and returned to its home cage. Throughout the survival times, the rats were kept inside a small safety cabinet. 

After a survival period of 3–21 days, the animals were deeply anaesthetized with sodium pentobarbital (100 mg/kg, i.p.) and were transcardially perfused and fixed with 10% sucrose in 0.1M phosphate buffer (PB; pH 7.4) followed by 4% paraformaldehyde in 0.1M PB. The brains were removed from the skulls, postfixed in the same fresh fixative for 4 hours at 4°C and then cryoprotected for at least 48 hours at 4°C in PB containing 30% sucrose. The brains were coronally sectioned at 50 μm on a freezing microtome. 

An immunofluorescence procedure similar to that used for the *in vitro* study was conducted to visualize RV-infected neurons. Fluorescent neurons were examined under a Zeiss Axiovert 200M, and imaged either with a laser scanning confocal unit (LSM 5 Exciter, Carl Zeiss) or with a digital camera (AxioCam MRm). 

To quantify the mRFP fluorescence intensity, the images of the infected cells were captured by using the laser scanning confocal microscope (LSM 5 Exciter, Carl Zeiss) under identical conditions. Since the ipsilateral hippocampal region contained numerous labeled neurons, the images were taken from the contralateral CA2, where only few rHEP5.0-ΔG-mRFP-infected neurons were observed in each slice. In the contralateral CA2 region of rHEP5.0-CVSG-mRFP-infected samples, there were many neurons labeled by the immunofluorescence of the N protein. Most of the infected neurons were considered to be trans-synaptically infected neurons because mRFP fluorescence was hardly observed in these neurons. In order not to underestimate the mRFP fluorescence intensity of the rHEP5.0-ΔG-mRFP-infected neurons, only few bright neurons in each slice, which were thought to be the initially infected neurons, were measured and used for quantitative analysis. Average intensity per pixel of mRFP fluorescence in the soma (arbitrary units) was measured using Zen software (Carl Zeiss). 

### Physiological recording in slice preparations

800 nl of rHEP5.0-ΔG-mRFP (1.0×10^8^ FFU/ml, N=8) was injected into the lateral septum (LS) of the 21- to 28-day-old rats as described above, and 3–16 days later the rats were anaesthetized with isoflurane and decapitated. Horizontal hippocampus slices (400 μm thick) were prepared using a vibratome (DTK-3000W, Dosaka, Japan) in ice-cold sucrose artificial cerebrospinal fluid (s-ACSF). The s-ACSF (pH 7.4) was composed of 185 mM sucrose, 2.5 mM KCl, 0.5 mM CaCl_2_, 25 mM NaHCO_3_, 1.2 mM NaH_2_PO_4_, 10 mM MgSO_4_, and 25 mM D-glucose oxygenated with a mixture of 95% O_2_-5% CO_2_ and chilled to 4°C. Before the start of recording, slices were submerged (in an incubation chamber) in standard ACSF at room temperature (26–28°C) for 1 h. The standard ACSF used for incubation and recording consisted of 119 mM NaCl, 2.5 mM KCl, 2 mM CaCl_2_, 25 mM NaHCO_3_, 1.2 mM NaH_2_PO_4_, 2 mM MgSO_4_, and 12.5 mM D-glucose oxygenated with a mixture of 95% O_2_-5% CO_2_ (pH 7.4). 

After 1 h of incubation in this chamber, a slice was transferred onto an experimental chamber placed on a fixed stage and subjected to the experiments. Neurons were visualized by oblique illumination with the aid of the contrast enhancement of a CCD-camera (C2741, Hamamatsu Photonics, Japan) mounted on an upright microscope (Axioskop 2FS, Ziess, Oberkochen, Germany). Infected cells were detected by direct visual observation of mRFP fluorescence. Patch-clamp recordings in the whole-cell mode were conducted as described above. Resting membrane potential was measured by taking the average voltage over 10 s in the absence of any current injection. Holding current maintaining the membrane potential at −80 mV was injected, and 500-ms current injections were used to elicit action potentials. 

### Statistics

Data shown in the Results section are expressed as mean values ± the SEM. The statistical significance of differences between means was evaluated by two-tailed Student’s t-testing and one-way ANOVA followed by Bonferroni’s post hoc test, both performed using Prism (Graphpad Software Inc., San Diego, CA).

## Results

### 1. Spread of viral vector in cultured cells

The infection characteristics of rHEP5.0-ΔG-mRFP were examined in NA cells and compared with those of rHEP5.0-CVSG-mRFP. NA cells were infected with either rHEP5.0-ΔG-mRFP or rHEP5.0-CVSG-mRFP to achieve 1–5% infection at 24 hours after infection and were fixed at different time points (1–6 days post-infection (dpi)). The infected cells were visualized by the immunofluorescence of viral N protein (Figure 1B). 

The number of cells infected with rHEP5.0-CVSG-mRFP increased significantly (P < 0.05, one-way ANOVA; post-hoc t-test with 1 dpi vs 3 dpi and 3 dpi vs 6 dpi, Bonferroni-corrected, all p < 0.016), and almost all cells were infected at 6 days post-infection (Figure 1C). The number of cells infected with rHEP5.0-ΔG-mRFP, in contrast, did not increase, and there were no significant differences in the numbers of infected neurons on days 1, 3, and 6 dpi (P = 0.26, one-way ANOVA).

### 2. Transgene expression level and cytotoxicity of viral vector in cultured cells

**Figure 2:**
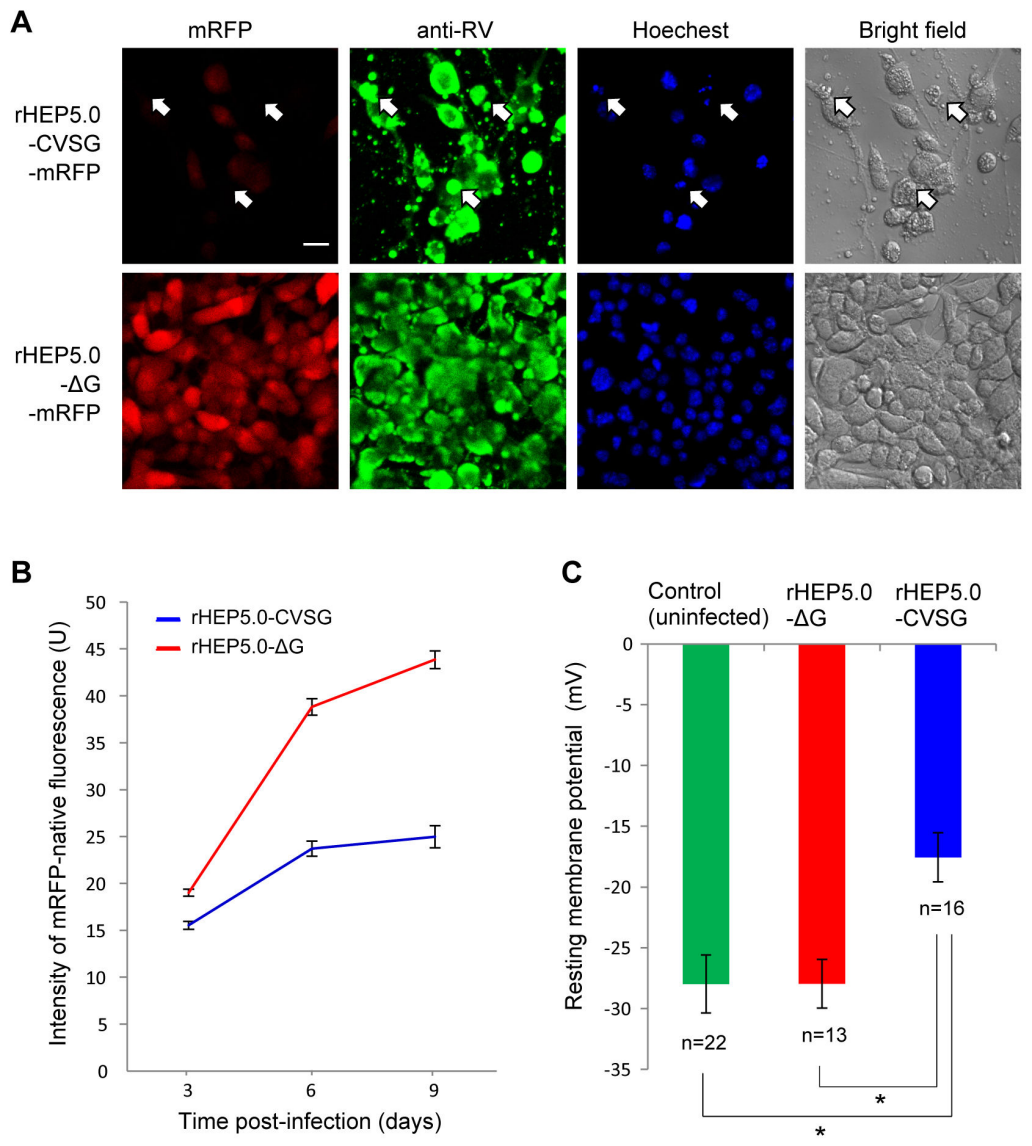
Characteristics of RV vectors in cultured cells. **A**: Photomicrographs of RV-vector-infected NA cells 9 dpi. Infection of the viral vector can be confirmed by immunofluorescence of the N protein (green). Note that rHEP5.0-ΔG-mRFP-infected cells show stronger mRFP fluorescence (red) than rHEP5.0-CVSG-mRFP-infected cells. The arrows show cells with fragmented nucleus that was revealed by the Hoechst staining (blue). Scale bar = 20 μm. **B**: Fluorescence intensity of infected cells (mean ± SEM) at different post-infection times. **C**: Resting membrane potential (mean ± SEM) of infected cells and uninfected control cells at 4 dpi, when the resting membrane potential of rHEP5.0-CVSG-mRFP-infected cells is significantly (*p < 0.016) higher than that of the rHEP5.0-CVSG-mRFP-infected cells and that of the control cells.

The mRFP expression level of rHEP5.0-ΔG-mRFP and rHEP5.0-CVSG-mRFP were compared in NA cells. Cells were infected with either rHEP5.0-ΔG-mRFP or rHEP5.0-CVSG-mRFP to achieve 90–100% infection at 24 hours after infection, and the fluorescence intensity of mRFP was examined at different time points (3, 6, and 9 dpi, Figure 2A, B). Three days after infection the mean fluorescence intensity of the rHEP5.0-ΔG-mRFP-infected cells was significantly higher than that of the rHEP5.0-CVSG-mRFP-infected cells (P < 0.0001, unpaired t test), and the intensity difference increased with the survival time. This result indicates that mRFP expression level of rHEP5.0-ΔG-mRFP is higher than that of rHEP5.0-CVSG-mRFP. 

There was also a difference in the number of attached cells between the dishes infected with these two viruses after long time periods (Figure 2A). Although at 3 days post-infection the numbers of cells in the two dishes were the same (P = 0.43, unpaired t-test), at 9 days post-infection the number of cells in the rHEP5.0-CVSG-mRFP-infected dish was significantly smaller than that in the rHEP5.0-ΔG-mRFP-infected dish (P < 0.0001, unpaired t-test). There was also a difference in the appearance of cells, and the rHEP5.0-CVSG-mRFP-infected cells appeared to have abnormally rough surfaces (Figure 2A). Furthermore, the fragmented nuclei characteristic of apoptosis [20] were observed in some cells infected with rHEP5.0-CVSG-mRFP (Figure 2A). In these cells, mRFP fluorescence was hardly observed. In contrast, there were apparently no differences between the control cells and the cells infected with rHEP5.0-ΔG-mRFP. 

We further examined the effect of viral infection on electrophysiological properties *in vitro*. NA cells were infected with either viral vector rHEP5.0-ΔG-mRFP or rHEP5.0-CVSG-mRFP, and the resting membrane potential was examined in each NA cell by using the whole-cell patch-clamp technique at different time points. Two days after infection the resting potential of the uninfected control cells (−26.2 ± 2.2 mV; n = 11) did not differ significantly from that of the cells infected with rHEP5.0-ΔG-mRFP (−28.0 ± 2.2 mV; n = 7) or that of the cells infected with rHEP5.0-CVSG-mRFP (−30.9 ± 3.8 mV; n=8) (P = 0.53, one-way ANOVA). Four days after infection, however, the resting membrane potential of the rHEP5.0-CVSG-mRFP-infected cells (−17.6 ± 2.0 mV, n = 16) was significantly higher than that the rHEP5.0-ΔG-mRFP-infected cells (−28.0 ± 2.0 mV; n=13) and that of the uninfected control cells (−28.0 ± 2.4 mV; n =2 2) (P < 0.05, one-way ANOVA; post-hoc t test, Bonferroni-corrected, P < 0.016 for control vs rHEP5.0-CVSG-mRFP infection and rHEP5.0-ΔG-mRFP infection vs rHEP5.0-CVSG-mRFP infection, Figure 2C). Furthermore, most of the rHEP5.0-CVSG-mRFP-infected cells showed much lower input resistance than the control cells and rHEP5.0-ΔG-mRFP-infected cells, which suggests that the viruses have caused cellular deterioration by this time. These histological and electrophysiological results show that the cytotoxicity of rHEP5.0-ΔG-mRFP is lower than that of rHEP5.0-CVSG-mRFP.

### 3. Expression level of viral vector *in vivo*

We next examined the mRFP expression level of rHEP5.0-ΔG-mRFP and rHEP5.0-CVSG-mRFP *in vivo* by injecting the viral vectors into the medial entorhinal cortex (MEC) of the rat. Ten rats received injections of rHEP5.0-ΔG-mRFP, and the intensity of mRFP fluorescence was examined at 3 dpi (n = 2), 5 dpi (n = 2), 7 dpi (n = 2), 14 dpi (n =2), and 21 dpi (n =2). Five rats received injections of rHEP5.0-CVSG-mRFP and were examined at 3 dpi (n = 2), 5 dpi (n = 1), and 7 dpi (n = 2).

**Figure 3:**
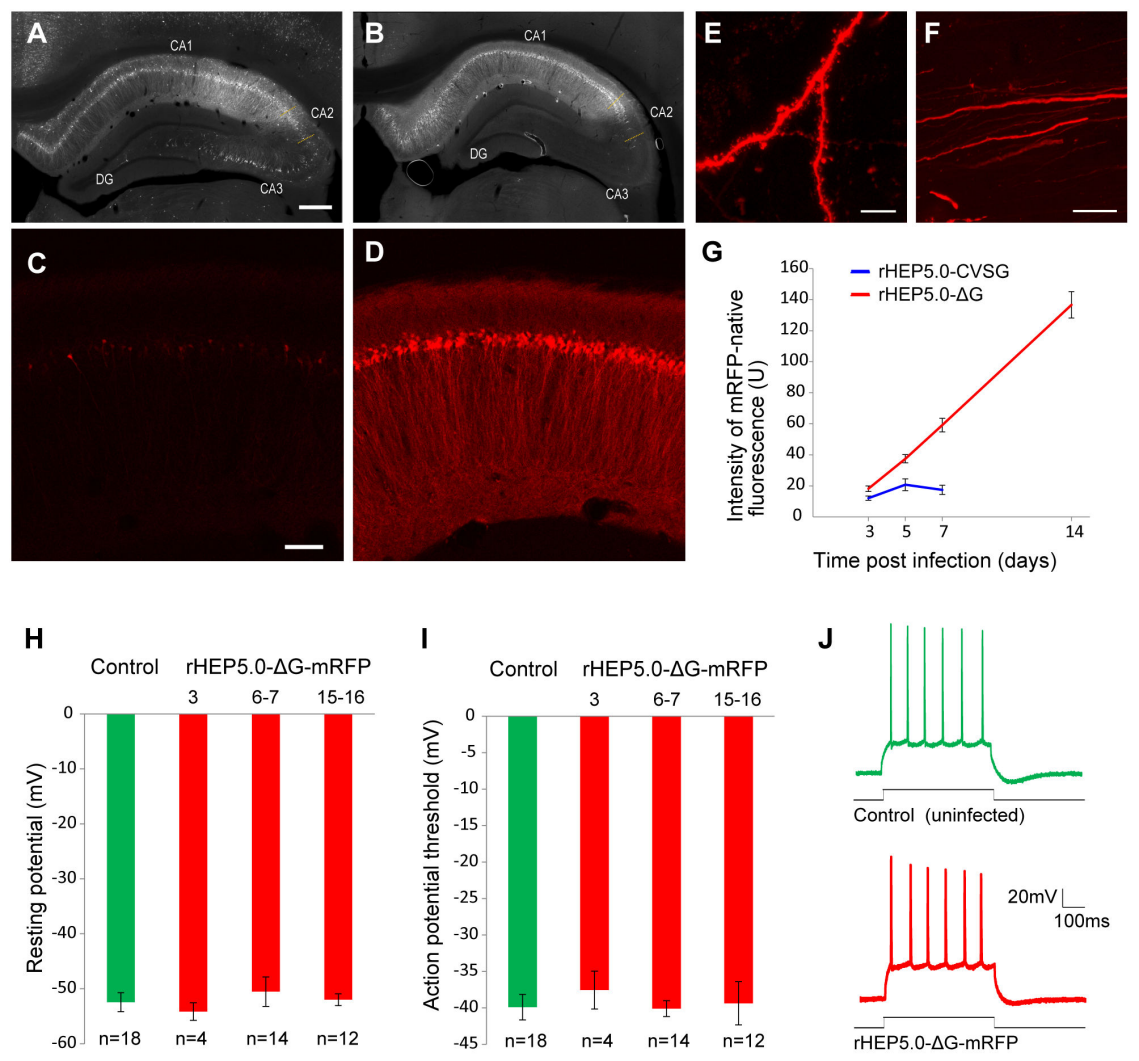
Characteristics of RV vectors *in* *vivo* and in brain slices. **A**–**D**: Fluorescence photomicrographs demonstrating immunoreactivity against N protein (A, B) and fluorescence of mRFP (C, D) in the dorsal hippocampus 7 days after injecting rHEP5.0-CVSG-mRFP (A, C) or rHEP5.0-ΔG-mRFP (B, D) into the MEC. **E**, **F**: High-magnification photomicrographs demonstrating Golgi-like labeling of rHEP5.0-ΔG-mRFP infected neurons. Dendritic spines were labeled at 3 dpi (E), and axons in the fimbria of the hippocampus were visualized at 5 dpi (F). Scale bar = 500 μm in (A) [applies to (B)], 100 μm in C [applies to (D)], 10 μm in (E), 20 μm in (F). **G**: Fluorescence intensity (mean ± SEM) of infected CA2 neurons at different post-infection times. **H**, **I**: Resting membrane potential (H) and action potential threshold (I) were recorded from the rHEP5.0-ΔG-mRFP-infected neurons and non-infected control neurons in hippocampal brain slices. **J**: Traces representative of membrane potentials in response to current injection (20 pA, 500 ms).

In all samples, many labeled neurons were observed in the subiculum, CA1, CA2, presubiculum, medial septum and the diagonal band, supramammillary nucleus, claustrum, midline dorsal thalamic nuclei, and lateral and anterior groups of the dorsal thalamus. These brain areas are all known to have direct projections to the MEC [21]. In samples infected with rHEP5.0-CVSG-mRFP, numerous labeled neurons were also observed in other brain regions, such as the hippocampal CA3 region (Figure 3A). Since neurons in CA3 project to the MEC indirectly by way of CA1, these labeled neurons are considered to be trans-synaptically labeled via CA1 neurons [7,22]. In rHEP5.0-ΔG-mRFP-infected samples, only a few labeled neurons were observed in the CA3 region (Figure 3B). Since the injection sites, which were determined by the presence of pontamine sky blue, were close to or in the angular bundle, we think that the sparse labeling of CA3 is a false positive result due to the viral uptake from the passing fibers. Indeed, similar labeling patterns in CA3 were seen in samples with injection of a non-trans-synaptic retrograde tracer (Fluoro-Gold) at the same coordinates (data not shown). These results suggest that rHEP5.0-ΔG does infect neurons retrogradely but does not propagate to the presynaptic neurons trans-synaptically.

To evaluate the ability of the viral vectors to express transgenes, we compared the native fluorescence of mRFP in the hippocampal region. Although 7 days after infection many CA1 neurons in both samples were infected (Figure 3A, B), rHEP5.0-ΔG-mRFP-infected cells showed much stronger fluorescence than rHEP5.0-CVSG-mRFP-infected cells (Figure 3C, D). In rHEP5.0-ΔG-mRFP-infected samples, intensive fluorescence labeling were observed not only in the pyramidal cell layer but also in other layers, including the alveus, which shows that dendrites and axons of the infected cells were strongly labeled (Figure 3D). The dendritic spines in some neurons were observed even at 3 dpi (Figure 3E), and the axons running in the fimbria of the hippocampus were clearly visualized at 5 dpi (Figure 3F). Similar mRFP fluorescence differences between these two vectors were also observed in other brain regions (data not shown). 

To quantify the difference in the transgene expression level of the two vectors, we measured the mean native fluorescence intensity from the soma of the infected contralateral CA2 neurons (Figure 3G). Three days after infection the mean fluorescence intensity of the rHEP5.0-ΔG-mRFP-infected neurons (18.2 ± 1.8 in arbitrary units; n = 20) was significantly (P < 0.05, unpaired t test) higher than that of the rHEP5.0-CVSG-mRFP-infected neurons (12.1 ± 1.4; n =20). The difference became more significant at longer survival times (P < 0.005 at 5 dpi, P < 0.0001 at 7 dpi, unpaired t test), and at 7 dpi the mRFP fluorescence intensity of rHEP5.0-ΔG-mRFP-infected neurons was 3.4 times higher than that of rHEP5.0-ΔG-mRFP-infected neurons. Fourteen days after rHEP5.0-ΔG-mRFP infection, detailed morphological features of the infected neurons were clearly visualized with strong mRFP labels. At 21 dpi, however, infected neurons showed cellular degeneration with fragmentation of the neurites (data not shown).

### 4. Cytotoxicity of rHEP5.0-ΔG-mRFP in slice preparation

To test whether rHEP5.0-ΔG can be used for physiological studies, rHEP5.0-ΔG-mRFP was injected into the lateral septum of the rat, and 3–16 days later the electrophysiological properties of the infected neurons in hippocampal brain slices were examined using whole-cell patch-clamp recording.

Many retrogradely labeled mRFP positive neurons were observed in the pyramidal cell layer of the CA1 and the subiculum. These fluorescent labeled neurons were compared with nonfluorescent control neurons in the pyramidal cell layer. Neither the input resistance nor the input capacitance differed significantly between the infected and non-infected neurons. Mean resting membrane potential was almost identical (P = 0.82, one-way ANOVA, Figure 3H) in control neurons (−52.5 ± 31.7 mV; n = 18) and rHEP5.0-ΔG-mRFP-infected neurons at 3 dpi (−54.2 ± 1.6 mV; n = 4), 6–7 dpi (−50.5 ± 2.7 mV; n = 14), and 15–16 dpi (−52.0 ± 1.1 mV; n = 12). There were also no significant differences between the action potential thresholds of infected neurons and control neurons (P = 0.96, one-way ANOVA, Figure 3I). Comparing the firing patterns elicited by whole-cell current injection (20 pA, 500 ms), we found that the infected and uninfected neurons showed similar patterns typically observed in the firing of hippocampal pyramidal cells (Figure 3J, [23]). 

## Discussion

In this study we have compared the properties of the propagation-competent RV vector (rHEP5.0-CVSG-mRFP) and the G-deleted RV vector (rHEP5.0-ΔG-mRFP) and have demonstrated that the deletion of G gene not only affects the infection property but also markedly affects the transgene expression level and the cytotoxicity of the RV vector. The glycoprotein of RV forms spike-like projections on the virus particle that are required for attachment to neuronal receptors and for viral entry into the cells. It has been reported that cells infected with the G-deleted virus were unable to produce infectious virus particles [24] and thus could not propagate to other neurons trans-synaptically [15]. In line with the previous studies, rHEP5.0-ΔG-mRFP did retrogradely infect neurons having direct projection to the injection site but did not show successive propagation to other synaptically connected neurons. This infection property of the G-deleted RV vector enables us to psuedotype the virus with an envelope protein of the avian sarcoma and leucosis virus (EnvA). This EnvA-pseudotyped G-deleted RV vector can be used to genetically target initial infection to particular neurons [13,16] because the infection by this pseudotyped virus would be restricted to the particular neurons which are engineered to express the cognate viral receptor (TVA). Furthermore, by supplying the G gene *in trans* within these initially infected neurons, it is also possible to control the trans-synaptic propagation of the RV vector and allow the vector to trans-synaptically infect the presynaptic neurons of the initially infected neurons. These infection features of the G-deleted virus enable a far more detailed understanding of the neural circuits than is possible with other viral vectors such as the adeno-associated virus or the lentivirus [25,26].

The RV glycoprotein is known to strongly affect the viability of infected cells because the accumulation of the glycoprotein at the cytoplasmic membrane is an important factor triggering apoptosis [20,27-29]. To our knowledge, however, there is no study that examined the effect of G deletion on the cytotoxicity of RV vector. In this study we have shown that rHEP5.0-CVSG-mRFP affects the resting membrane potential of the cultured cells 4 days after infection, whereas rHEP5.0-ΔG-mRFP does not (Figure 2C). We have further shown *in vivo* that infection with rHEP5.0-ΔG-mRFP does not influence the basic physiological properties of the infected neurons for at least 16 days after infection (Figure 3H–J). These results indicate that the deletion of G gene greatly reduces the cytotoxicity of the RV vector and enables us to maintain the physiological properties of the infected neurons approximately 4 times longer. 

One of the notable results in this study is the clear difference in the transgene expression levels of the two vectors. Because glycoprotein has little known involvement in genome transcription and replication, we expected that the G-deleted RV vector and the propagation-competent RV vector would show similar transgene expression levels. rHEP5.0-ΔG-mRFP, however, showed a mRFP expression level much higher than rHEP5.0-CVSG-mRFP did. The ability to expressing transgenes at a high level in a short period makes this G-deleted RV vector a useful tool for both anatomical and physiological studies. As we showed in this study, the fluorescence intensity of mRFP expressed by rHEP5.0-ΔG-mRFP was high enough to clearly visualize the infected neurons 3 days after infection (Figure 3E). Using this vector thus eliminates the need to wait 2–4 weeks to obtain sufficient transgene expression, as one must when using viral vectors such as the adeno-associated virus or the lentivirus [25,26,30,31]. 

There are two possible explanations for the mRFP expression level of rHEP5.0-ΔG-mRFP being higher than that of rHEP5.0-CVSG-mRFP. One is that it may be due to the differences in the cytotoxicity of the two viral vectors. Since rHEP5.0-CVSG-mRFP apparently affects the cell viability, the vector may cause some dysfunction in the transcription and replication system of the infected cells. In contrast, a lower cytotoxicity of rHEP5.0-ΔG-mRFP may realize longer-term expression of the transgene resulting in higher gene expression. Indeed, the mRFP expression level of rHEP5.0-ΔG-mRFP steadily increased at least for 14 days after infection whereas that of rHEP5.0-CVSG-mRFP did not increase after 5 days of infection (Figure 3G). The other possible explanation is that it may be due to the differences in the expression level of the L gene, which encodes the viral polymerase. In nonsegmented negative-strand RNA viruses, including *Rhabdoviridae*, the major element of transcriptional regulation is the gene order [32-34]. The viral RNA polymerase is assumed to enter the genome at the 3’ end and sequentially transcribe a leader RNA and monocistronic mRNAs. However, it is known that the transcription of genes attenuates at each gene border (transcriptional stop/start signal) because the polymerase dissociates from the template at each stop/polyadenylation signal and re-initiates poorly at the next start signal. It is also known that the size of the intergenic region, which is a non-transcribed sequence separating the stop/polyadenylation and start signal, can affect the transcription level [35]. The two viral vectors used in this study differ in the number of these border sequences. Since pHEP5.0-ΔG was generated by deleting the whole transcription unit of the G gene and the subsequent 2-nucleotide intergenic region from pHEP5.0-CVSG, rHEP5.0-ΔG-mRFP has fewer start and stop/polyadenylation signals and intergenic regions than rHEP5.0-CVSG–mRFP (Figure 1A). Because of this difference, it is presumed that the L gene, which is located at 5’-terminal, would be expressed at a higher level in rHEP5.0-ΔG-mRFP-infected cells than in rHEP5.0-CVSG-mRFP-infected cells. This higher expression of the polymerase might have resulted in a higher level of the mRFP expression as was shown in the previous study by Finke et al. [35]. Although which factor resulted in the increase of mRFP expression level in rHEP5.0-ΔG-mRFP is not known, Wickersham et al. [36] have recently reported that the insertion of additional transgene before the L gene does not drastically attenuate the expression of green fluorescent protein (EGFP) in SADΔG which has the EGFP gene in place of the G gene. This may indicate that the reduction of toxicity rather than the increase of L expression is responsible for the increased mRFP expression level in rHEP5.0-ΔG-mRFP.

The G-deleted RV vector reported in this study (rHEP5.0-ΔG) is different from SADΔG with respect to the RV strain and the position of the transgene insertion site [5-7,9,10]. In spite of these differences, the characteristics of rHEP5.0-ΔG are similar to those reported for SADΔG. It has been reported that SADΔG-infected neurons can be detected in a few days by the high-level expression of EGFP [9]. The intensity of EGFP labeling increases steadily after infection, and no widespread mortality is found until 16 days after infection [9,12]. These similarities of rHEP5.0-ΔG and SADΔG suggest that the mechanism underlying the high-level gene expression and low toxicity might be the same in the two vectors. This interesting mechanism remains to be clarified. These vectors have a great advantage for the studies which require a high-level of transgene expression in a short time and/or neuronal cell function for two to three weeks after an infection of the virus. 
